# Inhibition of miR-19a-3p decreases cerebral ischemia/reperfusion injury by targeting IGFBP3 in vivo and in vitro

**DOI:** 10.1186/s40659-020-00280-9

**Published:** 2020-04-20

**Authors:** Zhaohui Chai, Jiangbiao Gong, Peidong Zheng, Jiesheng Zheng

**Affiliations:** Department of Neurosurgery, The First Affiliated Hospital, College of Medicine, Zhejiang University, No. 79 Qingchun Road, Hangzhou, 310003 China

**Keywords:** Ischemic stroke, Ischemia/reperfusion injury, miR-19a-3p, IGFBP3

## Abstract

**Background:**

Inflammation and apoptosis are considered to be two main factors affecting ischemic brain injury and the subsequent reperfusion damage. MiR-19a-3p has been reported to be a possible novel biomarker in ischemic stroke. However, the function and molecular mechanisms of miR-19a-3p remain unclear in cerebral ischemia/reperfusion (I/R) injury.

**Methods:**

The I/R injury model was established in vivo by middle cerebral artery occlusion/reperfusion (MCAO/R) in rats and in vitro by oxygen–glucose deprivation and reperfusion (OGD/R) induced SH-SY5Y cells. The expression of miR-19a-3p was determined by reverse transcription quantitative PCR. The infarction volumes, Neurological deficit scores, apoptosis, cell viability, pro-inflammatory cytokines and apoptosis were evaluated using Longa score, Bederson score, TTC, TUNEL staining, CCK-8, ELISA, flow cytometry assays. Luciferase reporter assay was utilized to validate the target gene of miR-19a-3p.

**Results:**

We first found miR-19a-3p was significantly up-regulated in rat I/R brain tissues and OGD/R induced SH-SY5Y cells. Using the in vivo and in vitro I/R injury model, we further demonstrated that miR-19a-3p inhibitor exerted protective role against injury to cerebral I/R, which was reflected by reduced infarct volume, improved neurological outcomes, increased cell viability, inhibited inflammation and apoptosis. Mechanistically, miR-19a-3p binds to 3′UTR region of IGFBP3 mRNA. Inhibition of miR-19a-3p caused the increased expression of IGFBP3 in OGD/R induced SH-SY5Y cells. Furthermore, we showed that IGFBP3 overexpression imitated, while knockdown reversed the protective effects of miR-19a-3p inhibitor against OGD/R-induced injury.

**Conclusions:**

In summary, our findings showed miR-19a-3p regulated I/R-induced inflammation and apoptosis through targeting IGFBP3, which might provide a potential therapeutic target for cerebral I/R injury.

## Background

As the most common type of stroke, ischemic stroke is characterized by the sudden loss of blood circulation to an area of the brain, which represents a major public health problem [1]. Currently, rapid restoration of the blood supply has been the most effective treatment for ischemic stroke. However, further brain injury and dysfunction following ischemia may be aggravated by blood reperfusion, which is known as cerebral ischemia/reperfusion (I/R) injury [2]. Therefore, it urgently needed to elaborate the underlying molecular mechanisms to improve the functional recovery after cerebral I/R injury.

According to an increasing number of studies, the mechanisms of cerebral I/R injury are complex, of which the inflammation and apoptosis are considered to be main factors inducing nerve cell injury after I/R [3–6]. It is known that microRNAs (miRs), as small non-coding RNA molecules (19–24 nts), could modulate diverse biological processes, including cell proliferation, apoptosis and neuroinflammation through binding to the 3′-UTR region of their target mRNA [7–9]. With the development of ischemic stroke studies, investigation of the role of miRs in cerebral I/R injury has been increased. For example, miR-132 has been reported to attenuate cerebral injury by protecting blood–brain barrier disruption in ischemia stroke [10]. MiR-224-3p may protect N2a cells from cerebral I/R injury by targeting FAK family-interacting protein (FIP200) [11]. On the contrary, miR-27b inhibition promotes recovery after ischemic stroke by regulating AMP-activated protein kinase (AMPK) activity [12]. All the above-mentioned reports strongly suggest that miRs play an important role in the process of I/R injury. Recently, the broadly conserved miR-19a-3p, a crucial component of the miR-17-92 cluster, has been shown to be a mediator of the cell proliferation-inhibitory effect in breast cancer [13], cell apoptosis in chemosensitivity of osteosarcoma [14] and inflammatory responses [15]. Interestingly, our attention was aroused by miR-19a-3p which is the most widely modulated miRNA as novel biomarker in ischemic stroke by Eyileten et al. [16]. However, the possible mechanisms of miR-19a-3p against inflammation and apoptosis in cerebral I/R injury are still understudied.

Insulin-like growth factor-1 (IGF-1) is a mediator of growth hormone that promotes human growth by directly acting on the growth hormone receptor [17]. As the main binding protein of IGF-1, insulin-like growth factor binding protein-3 (IGFBP3) has been reported to be linked to pathogenesis of cancers by exerting tumor suppressor activity in breast cancer [18] and pro-tumor effects in oral squamous cell carcinoma [19] and lung cancer [20]. According the report by Krakowska-Stasiak et al. [21], the levels of IGF‑1/IGFBP3 were lower in patients with inflammatory bowel disease. Notably, IGF-I and IGFBP-3 concentrations after acute cerebral ischemia were strikingly lower than those in control subjects and healthy individuals reported in Schwab et al. [22], Denti et al [23], Johnsen et al. [24]. These evidences indicate IGFBP-3 might present neuroprotective effects against cerebral I/R injury.

In this study, we investigated the role of miR-19a-3p in inflammation and apoptosis in middle cerebral artery occlusion (MCAO) rat model and in vitro oxygen and glucose deprivation/reoxygenation (OGD/R) induced SH-SY5Y cell model. Moreover, a new target IGFBP3 of miR-19a-3p was identified using bioinformatics software and validated by luciferase reporter assay. Furthermore, we further provided direct evidence that miR-19a-3p regulated OGD-induced SH-SY5Y cell injury by targeting IGFBP3. Our findings might provide a new insight into the mechanism of cerebral I/R injury.

## Materials and methods

### Animal groups

Healthy male Sprague-Dawley rats, weighing 200–250 g, were purchased from the Experimental Animal Center of College of Medicine, Zhejiang University (Zhejiang, China). Rat were housed in standard cages (22–25 °C and 45–50% humidity) with 12-h light/dark cycle and allowed free access to food and water. Rats were randomly divided into the following three groups (n = 6 each group): (1) Sham group; (2) Middle cerebral artery occlusion (MCAO) group; (3) MCAO + inhibitor group, in which miR-19a-3p inhibitor (GCTCAAACTGTTTATCTTCCATGCGAGTTTG) was a chemically synthesized exogenous miR-19a-3p mature sequence inhibitor supplied by GenePharma Co., Ltd. (Shanghai, China) and diluted with EntransterTM in vivo transfection reagent (Engreen, Beijing, China). Then, rats were administered intracerebroventricular injection of miR-19a-3p inhibitor using a microsyringe (Hamilton, Nevada, USA) 3 days prior to MCAO. All animal experiments were carried out in this study were approval by the Ethics Committee of The First Affiliated Hospital, College of Medicine, Zhejiang University (No. 2018-642, Date: 20180516) and followed the guidance of the National Institutes of Health Guide for the Care and Use of Laboratory Animals (No. 80-23, revised 1996).

### MCAO treatment

Rat model of cerebral I/R injury was established by 2 h of MCAO via intraluminal filament method as described previously [25]. Briefly, rats were subcutaneously anesthetized with chloral hydrate (400 mg/kg, ip) and then placed in the supine position on operating table. The right common carotid artery, external and internal carotid arteries were exposed by a midline skin incision. Then, a heparinized intraluminal filament with rounded tip (diameter 0.22 ± 0.02 mm) was inserted from external carotid artery through the internal carotid artery to reach the MCA to block the origin of MCA. After operation, the surgical site was sutured and the filament was withdrawn, followed by 24 h of reperfusion. The rats in the sham group had the same surgery except that the intraluminal filament was not inserted to the MCA origin. Brain infarct volumes, neurological scores and TUNEL staining were evaluated at 72 h after reperfusion.

### 2, 3, 5-Triphenyltetrazolium chloride (TTC) staining

TTC staining was performed to histologically verify the success of the model. In brief, the brains tissues from different groups were collected and frozen for 30 min at − 20 °C. Then, the brain tissues were sliced into 2-mm-thick sections and incubated with 2% TTC solution (Sigma-Aldrich, St. Louis, MO, USA) at 37 °C for 20 min, which was terminated by rinsing with PBS. Subsequently, the slice section was fixed with 4% paraformaldehyde for 2 h and photographed. The infarct volume was expressed as a percentage of total infarct volume/total brain volume × 100% by Image-Pro Plus 6.0 analysis software.

### Neurological deficit evaluation

Neurological deficit evaluation was performed after reperfusion using modified Longa score [26] and Bederson score [27] by an assessor who was blinded to the experimental groups. The Longa score was assessed on a scale of 0 to 4: 0 = no observable deficits; 1 = failure to fully extend the left forepaw; 2 = difficulty in circling to the left; 3 = failing to the left side; 4 = no spontaneous walking with decreased level of consciousness. The Bederson score was graded on a 5-point scale as follows: 0 = no deficits; 1 = lost forelimb flexion; 2 = lost forelimb flexion with lower resistance to lateral push; 3 = unidirectional circling; 4 = longitudinal spinning or seizure activity; 5 = no movement. For Longa score and Bederson score, the higher the score is, the more severe the damage.

### TUNEL staining

The apoptosis of cortical neurons was determined using an in situ terminal deoxynucleotidyl-transferase-mediated 2′-deoxyuridine 5′-triphosphate nick-end labeling assay (TUNEL, Roche Diagnostics, Germany). Briefly, rat brain tissues in cortex region were fixed in 4% paraformaldehyde, cut into 14 μm coronal sections and stained as the manufacture’s instruction. Then, the sections were immerged into 4′, 6-diamidino-2-phenylindole (DAPI) staining (Beyotime Biotechnology, China). TUNEL-positive (green) and DAPI-positive (blue) staining patterns were acquired under a fluorescence microscope (Olympus IX71; Olympus Corporation, Tokyo, Japan).

### In vitro model of OGD/R

The in vitro model for simulating I/R injury was constructed by oxygen and glucose deprivation/reoxygenation (OGD/R). Here, we used a neuron-like human-derived neuroblastoma cell line SH-SY5Y, which was purchased from American Type Culture Collection (ATCC, Manassas, VA, USA). Cells in normoxia group were cultured in DMEM medium (HyClone, Logan, UT, USA) with 10% fetal bovine serum (FBS, Gibco, USA) and incubated at 37 °C in a humidified atmosphere containing 5% CO_2_. For OGD/R, cells were cultured for 8 h in culture medium with deprivation of glucose and serum in an oxygen-free condition at 37 °C. Subsequently, the cells were returned to normal medium under normoxic conditions to allow for reoxygenation for 24 h.

### Cell transfection

The miR-19a-3p inhibitor (inhibitor) and its negative control (miR-NC) were synthesized by GenePharma Co., Ltd. (Shanghai, China). The pcDNA3.1/IGFBP3 vector was generated by inserting the open reading frame of IGFBP3 without 3′UTR into the pcDNA3.1 vector (Sangon Biotech, Shanghai, China). Small interference RNA against IFGBP3 (si-IGFBP3) was purchased from GenePharma Co., Ltd. Before OGD/R, SH-SY5Y cells were seeded at a density of 3 × 10^5^ cells per well and transfected with inhibitor, miR-NC, IGFBP3 or empty vector. In the rescue experiments, si-IGFBP3 was transfected into miR-19a-3p silenced SH-SY5Y cells. All these transfection procedures were performed for 48 h using Lipofectamine 2000 (Invitrogen, Carlsbad, CA, USA) following the manufacturer’s protocols.

### Enzyme linked immunosorbent assay (ELISA)

Briefly, ischemic brain tissues from rats were harvested, homogenized, and centrifuged (12,000 rpm for 20 min) at 4 °C. Cell supernatants were collected from SH-SY5Y cells by centrifugation (12,000 rpm for 15 min). Then, the levels of TNF-α (EK0526 96T), IL-1β (EK0393), and IL-6 (EK0412 96T) in ischemic brains and cell supernatants were measured by using commercial ELISA kits (Boster Biosciences Co., Wuhan, China) according to the manufacturer’s instructions.

### Analysis of cell viability

Cells were seeded into 96-well plates at a density of 5 × 10^3^ cells per well and cultured overnight at 37 °C. Next day, pre-made Cell Counting Kit-8 solution (10 μL per well, Beyotime Biotechnology) was added to each well and cells were incubated for 1 h at 37 °C. The optical density values at 450 nm were measured by Microplate Reader (Bio-Rad, Hercules, CA, USA).

### Cell apoptosis analysis

Cell apoptosis was measured using Annexin V-FITC apoptosis detection kit (BD Biosciences, San Jose, CA) according to the manufacturer’s instructions. Briefly, approximately 5 × 10^4^ cells were collected, washed twice with PBS and subjected to Annexin V-FITC/PI double staining at room temperature for 20 min in the dark. The differentiation of apoptotic cells (Annexin V positive) was detected by flow cytometry (BD Bioscience, San Jose, CA).

### Reverse transcription quantitative PCR (RT-qPCR)

Total RNA of brain tissue or cultured cells was extracted using TRIzol reagent (Takara Biotechnology, Japan) and first-strand cDNA was synthesized using a Prime Script TM RT Reagent kit (Takara Biotechnology) according to the manufacturer’s instructions. MiR-19a-3p expression was determined using SYBR Premix Ex Taq™ II kit (Takara Bio, Inc., Otsu, Japan) with U6 as an internal reference. For IGFBP3 mRNA analysis, RT-qPCR was performed using a SYBR Green PCR Master Mix Kit (Applied Biosystems) with β-actin as an internal reference. All PCR reaction was conducted on a 7500 FAST Real-Time PCR System (Bio-Rad Co., USA) according to the amplification protocols consisting of an initial denaturation step at 95 °C for 10 min; 40 cycles of 95 °C for 1 min and 60 °C for 40 s, 72 °C for 30 s, and 72 °C for 1 min. The primers used were as following, miR-19a-3p forward, 5′-GGCGGGGAAAGTGTGTCT-3′, and reverse, 5′-GTGCAGTCGTGGCGTGTG-3′; U6, forward, 5′-GCACATATACGCTTCGGCATAAAAT-3′, and reverse, 5′-CATTTGCGGCTTCACGATGTCAT-3′; IGFBP3, forward, 5′-TGATGGATTGGCTCCTGAAATTATG-3′, and reverse, 5′-CTTGTTATCTGACAGGGAAGTGCCG-3′; β-actin, forward, 5′-TGTCACCAACTGGGACGATA-3′, and reverse, 5′-GGGGTGTTGAAGGTCTCAAA-3′. Data were analyzed with the 2^−ΔΔCt^ method and expressed as folds over experimental control groups.

### Western blot analysis

Total protein was extracted using the RIPA Lysis and Extraction Buffer and protein concentrations were measured by BCA Protein Assay reagent kit (both from Beyotime Biotechnology) according to the manufacturer’s protocol. Equal amounts of protein samples (30 μg) were separated by 10% sodium dodecyl sulfate–polyacrylamide gel electrophoresis (SDS-PAGE) and transferred onto PVDF membranes. The membranes were blocked with 5% non-fat dry milk in TBST for 2 h at room temperature and then incubated overnight at 4 °C with primary antibodies against IGFBP3, Bcl-2, Bax and GAPDH, followed by incubation with horseradish peroxidase-conjugated second antibody for 2 h at room temperature. Blots were visualized by an enhanced chemiluminescent substrate (Thermo, Fisher Scientific).

### Dual luciferase reporter assay

Prediction of the putative binding site between miR-19a-3p and IGFBP3 was conducted using TargetScan version 7.2 (http://www.targetscan.org/vert_72/). The 3′UTR of IGFBP3 mRNA containing miR-19a-3p binding sites (UUUGCAC) was amplified from cDNA of SH-SY5Y cells and inserted into pGL3 vector (Promega, Madison, WI, USA) to construct pGL3-IGFBP3 3′UTR-wild type (WT IGFBP3) by GenePharma Co., Ltd and cloned into pGL3 vector (Promega, Madison, WI, USA). Meanwhile, the pGL3-IGFBP3 3′UTR-mutant (MUT IGFBP3) with mutation of predicted miR-19a-3p binding sites was constructed by site mutation of WT IGFBP3 using a QuikChange Site-directed Mutagenesis kit (Agilent Technologies, Inc., Santa Clara, CA, USA). Subsequently, SH-SY5Y cells were co-transfected with 200 ng of the luciferase reporter plasmid and 50 nM of the miR-19a-3p inhibitor or miR-NC using Lipofectamine 2000 (Invitrogen). The firefly and renilla luciferase activity were measured after 48 h using dual luciferase reporter assay system (Promega). Relative luciferase activity was determined with renilla luciferase as normalization.

### Statistical analysis

All data were analyzed by SPSS 21.0 software (SPSS Inc., Chicago, IL, USA) and expressed as the mean ± SD. Values of *p* less than 0.05 were considered statistically significant. All quantitative data were analyzed using Student’s t tests for two groups, while one-way ANOVA followed by Tukey’s post hoc test for multiple comparisons.

## Results

### Down-regulation of miR-19a-3p protected rat brain against cerebral I/R injury

**Fig. 1:**
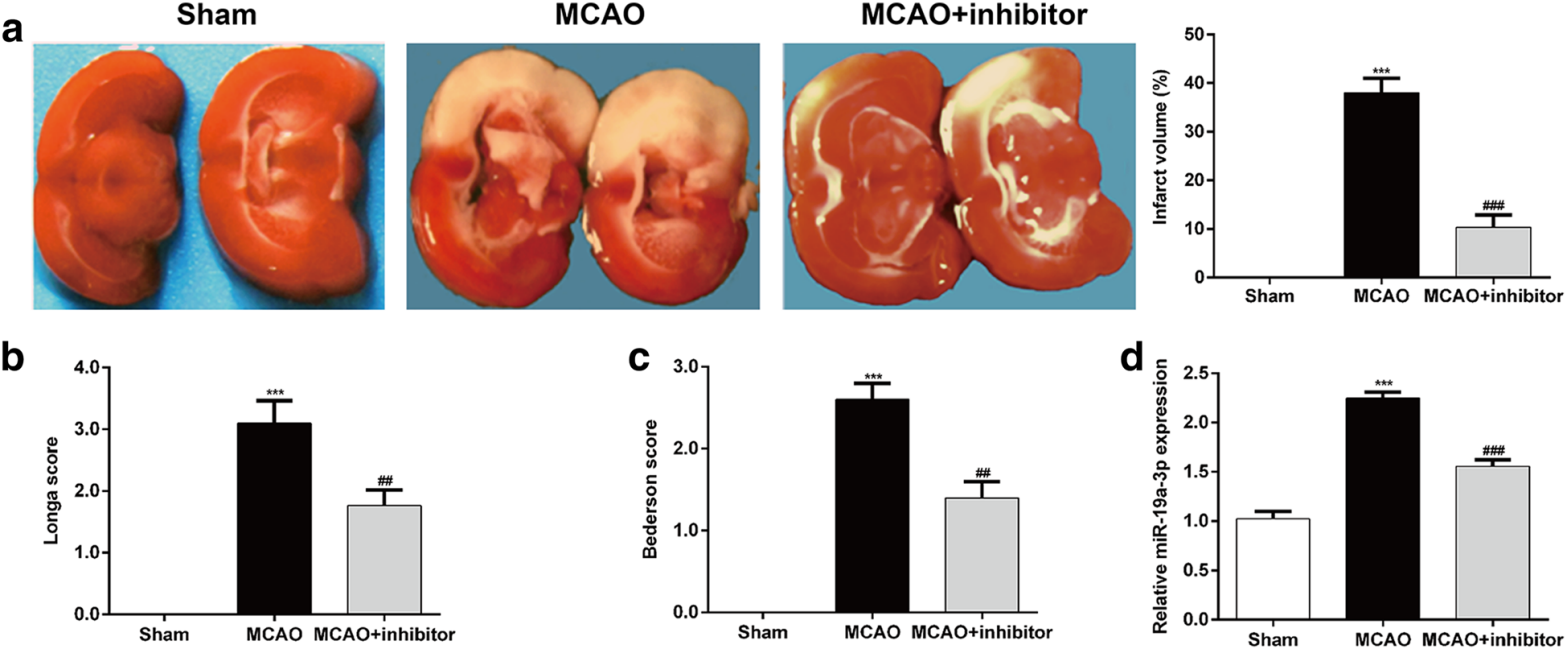
Down-regulation of miR-19a-3p exerted a neuroprotective role in focal cerebral ischemia/reperfusion rats. **a** Representative images of TTC staining in brain sections collected from rats in MCAO group receiving intracerebroventricular injection of miR-19a-3p inhibitor at 72 h after reperfusion (left panel). The relative infarct area percentage was evaluated by observing the unstained white infarcted tissue zone and the stained red normal tissue zone (right panel). **b** The Longa score (n = 6 per group) and **c** Bederson core (n = 6 per group) were applied to assess neurological function deficits. **d** The expression of miR-19a-3p in Sham rat brains and I/R rat brains treated with miR-19a-3p inhibitor was analyzed by RT-qPCR. The experiments were performed in triplicate and each value represented mean ± SD. ***p < 0.001, compared with Sham; ##*p* < 0.01, ###*p* < 0.001, compared with MCAO

To investigate the potential role of miR-19a-3p in brain I/R injury, the rats randomly received an intracerebroventricular injection of miR-19a-3p inhibitor prior to MCAO treatment, with Sham as control. As shown in Fig. 1a, the infarct region was obviously observed in the brain of MCAO groups compared with Sham group. However, the infarct volume was significantly reduced in MCAO treated with miR-19a-3p inhibitor. Meanwhile, the neurological function deficits in MCAO + inhibitor group were significantly improved compared to those in MCAO group in terms of the Longa score (Fig. 1b, n = 6 each group) and Bederson score (Fig. 1c, n = 6 each group). We further examined the expression of miR-19a-3p associated with injury of cerebral I/R. RT-qPCR analysis showed that the expression of miR-19a-3p was significantly increased in the MCAO group compared with Sham group, but notably decreased after intracerebroventricular injection of miR-19a-3p inhibitor in MCAO group (Fig. 1d). These data indicate that the injury of cerebral I/R could be improved by miR-19a-3p silence.

### Down-regulation of miR-19a-3p suppressed inflammation and apoptosis caused by I/R injury

**Fig. 2:**
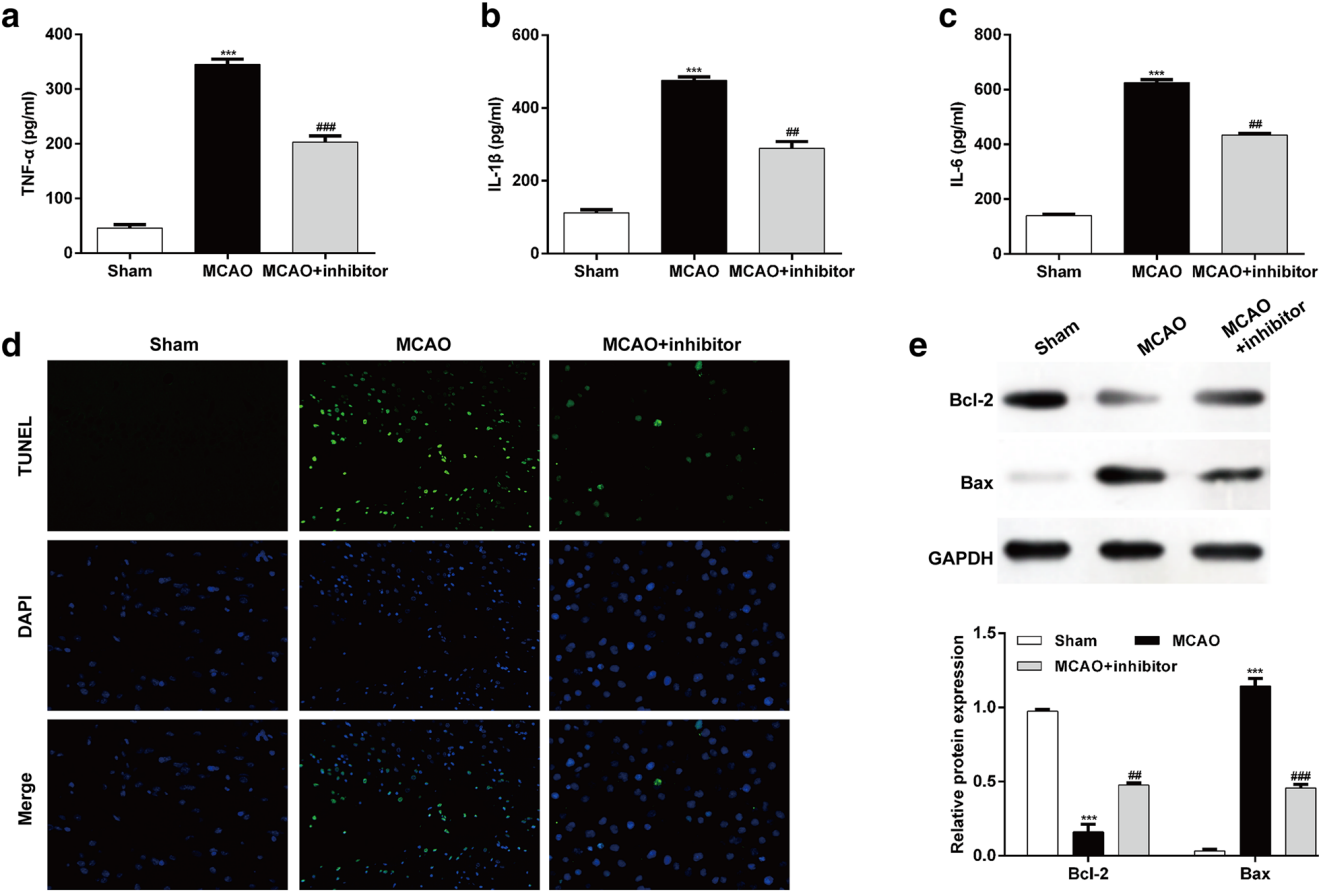
The effect of miR-19a-3p on pro-inflammatory cytokines and apoptosis in I/R rat brain. The levels of TNF-α **a**, IL-1β **b**, and IL-6 **c** in the Sham, MCAO, and MCAO + inhibitor groups were measured by ELISA (n = 6 per group). **d** The apoptosis of cortical neurons was evaluated by TUNEL staining. **e**–**f** Measurement of Bcl-2 and Bax protein levels in Sham, MCAO and MCAO + inhibitor groups using western blotting. The experiments were performed in triplicate and each value represented mean ± SD. ***p < 0.001, compared with Sham; ##*p* < 0.01, ###*p* < 0.001, compared with MCAO

To clarify the downstream mechanism of miR-19a-3p knockdown-mediated protection from injury of cerebral I/R, we analyzed the effects of miR-19a-3p knockdown on inflammation and apoptosis, known as the indication of I/R injury. ELISA assay showed that the massive production of pro-inflammatory cytokines, including TNF-α (Fig. 2a), IL-1β (Fig. 2b), and IL-6 (Fig. 2c) in MCAO group could be significantly decreased by injection of miR-19a-3p inhibitor. In addition, TUNEL assay showed more TUNEL-positive cells was observed in the brain sections from the MCAO group, whereas miR-19a-3p inhibitor treatment induced significant decrease in the TUNEL-positive cells in MCAO group, which was also reflected by the fluorescence intensity labeled by TUNEL staining (Fig. 2d). Furthermore, a significant decrease in anti-apoptotic Bcl-2 and increase in pro-apoptotic Bax were observed in MCAO group compared with those of the sham group. Significant elevation in the Bcl-2 expression and reduction in the Bax expression were noticed after miR-19a-3p knockdown in MCAO group (Fig. 2e). Collectively, these findings indicate that down-regulation of miR-19a-3p could exert protective role against cerebral I/R injury through suppressing inflammation and apoptosis.

### Down-regulation of miR-19a-3p protected SH-SY5Y cells against OGD/R-induced injury

**Fig. 3:**
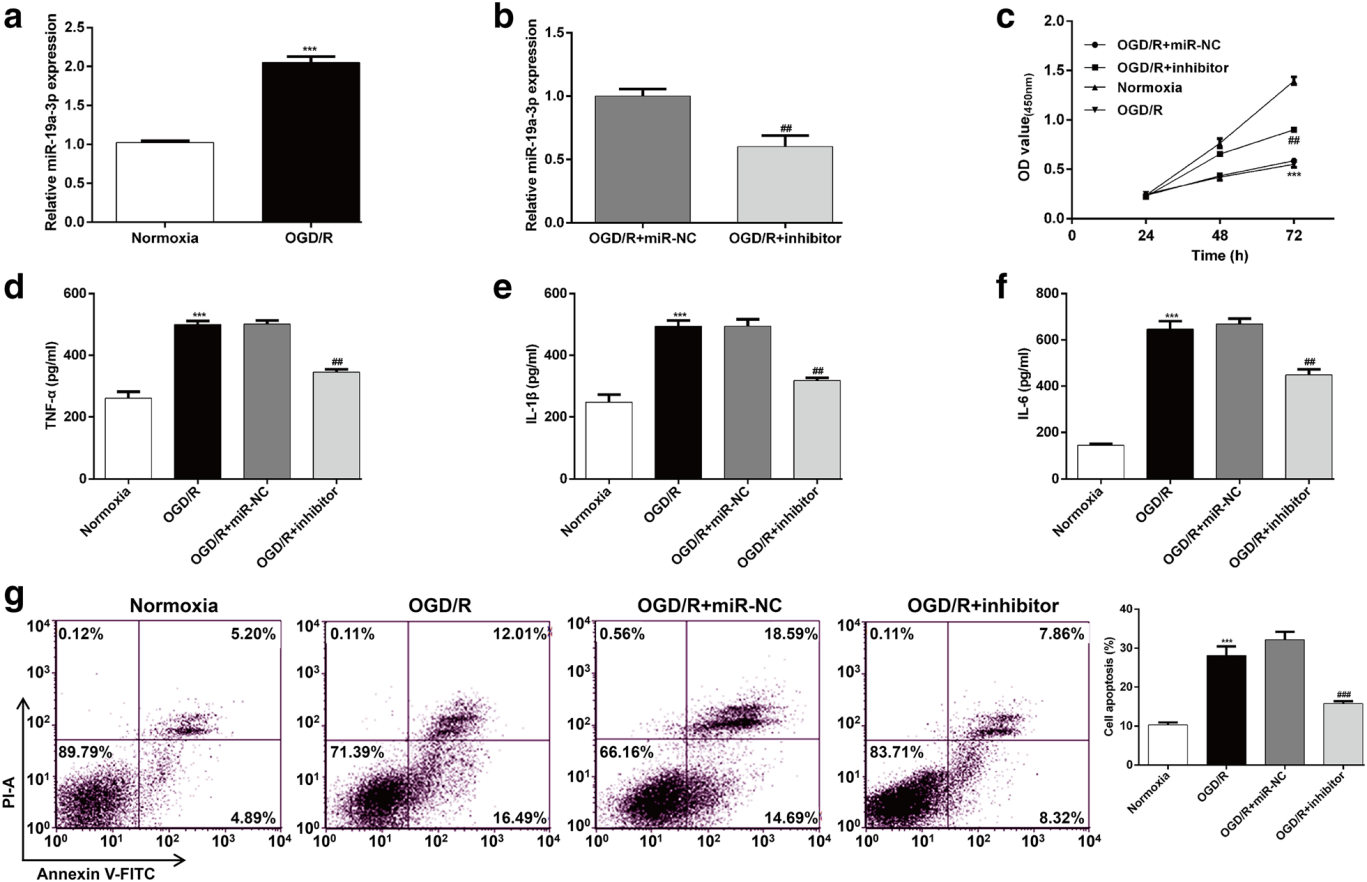
Down-regulation of miR-19a-3p protected SH-SY5Y cells against OGD/R-induced injury. SH-SY5Y cells were transfected with miR-19a-3p inhibitor or miR-NC, followed by OGD/R exposure. The expression of miR-19a-3p was determined in (**a**) Normoxia and OGD/R group, as well as (**b**) OGD/R + miR-NC and OGD/R + inhibitor group. **c** CCK-8 assay was utilized to analyze cell viability. The levels of TNF-α (**d**), IL-1β (**e**), and IL-6 (**f**) were measured by ELISA assay. **g** Cell apoptosis was determined with Annexin V/PI double staining followed by flow cytometry assay. The experiments were performed in triplicate and each value represented mean ± SD. ***p < 0.001, compared with Normoxia; ##*p* < 0.01, ###*p* < 0.001, compared with OGD/R + miR-NC

To determine the role of miR-19a-3p on cellular OGD/R injury, OGD/R cell model was established in SH-SY5Y cells. The expression of miR-19a-3p was measured using RT-qPCR. As shown in Fig. 3a, miR-19a-3p expression in OGD/R group was significantly higher than that in the normoxia group. Subsequently, miR-19a-3p inhibitor had been successfully transfected into the OGD/R cells, as demonstrated by remarkably reduced miR-19a-3p expression (Fig. 3b). Then the effects of miR-19a-3p on cell viability, inflammation and apoptosis were evaluated in OGD/R cell model. CCK-8 assay showed that cell viability was significantly reduced in OGD/R cells compared to those in normoxic cells, and that miR-19a-3p knockdown notably alleviated such decrease (Fig. 3c). On the contrary, the levels of TNF-α (Fig. 3d), IL-1β (Fig. 3e), and IL-6 (Fig. 3f) were significantly increased when SH-SY5Y cells were under OGD/R conditions. There observed increases were reversed posterior to the transfection of miR-19a-3p inhibitor into the OGD/R cells (Fig. 3d–f). In addition, OGD/R-induced SH-SY5Y cells exhibited notable increases in apoptotic cells population, which was partly abolished by miR-19a-3p silencing (Fig. 3g).

### IGFBP3 was a potential target of miR-19a-3p

**Fig. 4:**
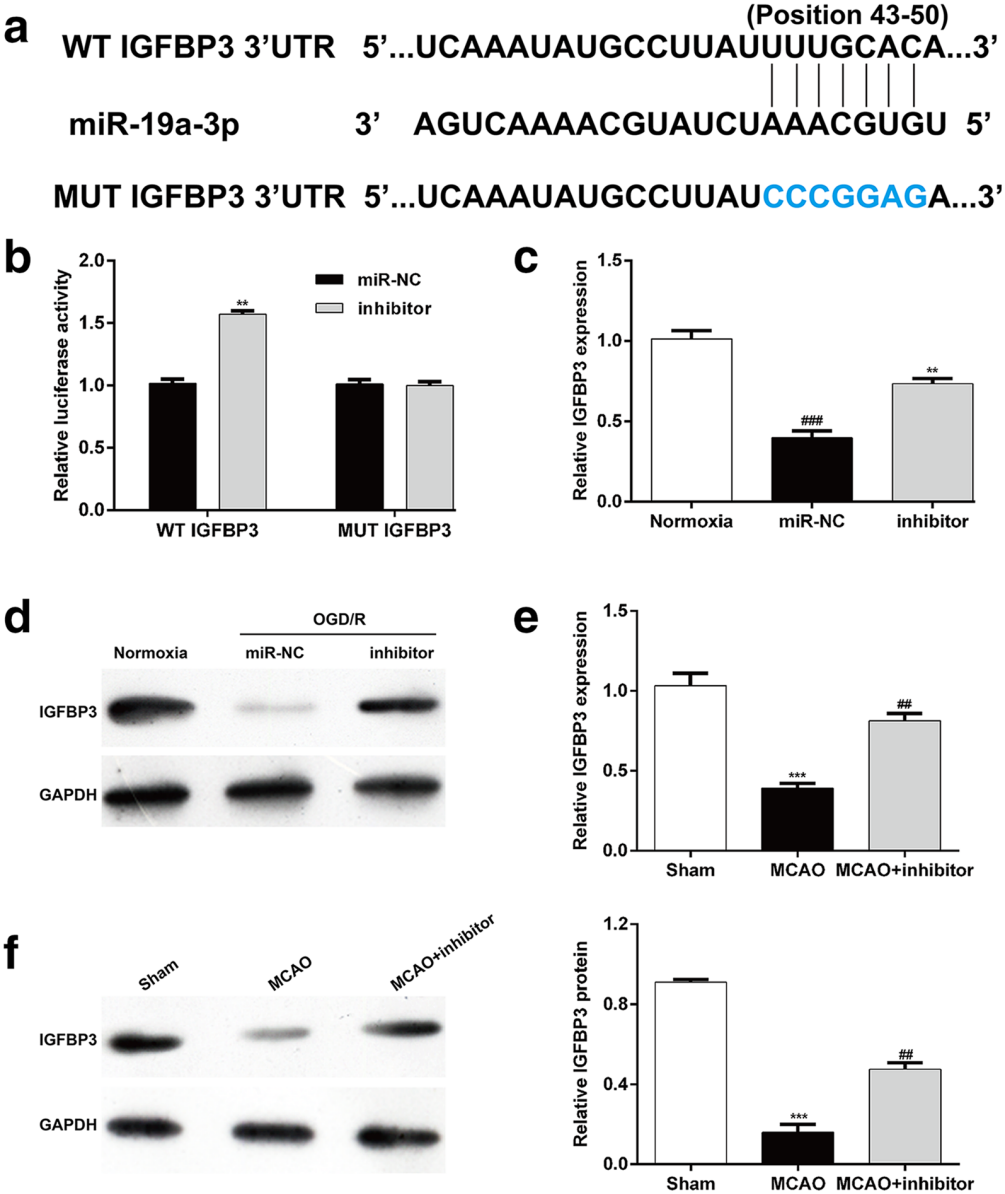
IGFBP3 3′-untranslated region (UTR) was directly targeted by miR-19a-3p. **a** Schema of the WT and mutated IGFBP3 3′-UTR indicating the interaction sites between miR-19a-3p and the 3′-UTR of IGFBP3. **b** Dual luciferase assay in SH-SY5Y cells co-transfected with the miR-19a-3p inhibitor and reporter vectors containing either the wild-type or mutated 3’-UTR of IGFBP3. **c** RT-qPCR and **d** Western blot analysis were used to determine the expression of IGFBP3 in SH-SY5Y cells transfected with miR-19a-3p inhibitor or miR-NC, and then was exposed to OGD/R conditions. **p < 0.01, compared with miR-NC; ###*p* < 0.001, compared with Normoxia; The expression levels of IGFBP3 mRNA (**e**) and protein (**f**) were determined in Sham rat brains and I/R rat brains treated with miR-19a-3p inhibitor. The experiments were performed in triplicate and each value represented mean ± SD. ***p < 0.01, compared with Sham; ##*p* < 0.01, compared with MCAO

To explore the mechanisms miR-19a-3p silencing modulated inflammation and apoptosis, we identified the potential gene targets of miR-19a-3p silencing by using the TargetScan program. Among all of the predicted gene targets, IGFBP3 was chosen as a candidate because it is reported to be associated with ischemic stroke. As shown in Fig. 4a, the potential binding site in the 3′-UTR of the IGFBP3 mRNA was identified as being targeted by miR-19a-3p. Then, dual luciferase reporter assay was performed to obtain the direct evidences that IGFBP3 was a target of miR-19a-3p. It was found that miR-19a-3p inhibitor significantly increased the luciferase activity in the SH-SY5Y cells transfected with the WT IGFBP3, but not in the cells transfected with the MUT IGFBP3 (Fig. 4b). Moreover, we further observed the down-regulated IGFBP3 mRNA and protein expression by OGD/R exposure was significantly reversed by miR-19a-3p inhibitor transfection in SH-SY5Y cells (Fig. 4c–d). In addition, we determined the expression of IGFBP3 in I/R injury rat model. The results showed that the expression of IGFBP3 mRNA and protein were significantly down-regulated in MCAO group compared with Sham group. Notably, miR-19a-3p inhibitor injection remarkably increased the expression of IGFBP3 mRNA and protein in MCAO group (Fig. 4e–f). These results suggested that miR-19a-3p likely binds to the 3′-UTR of IGFBP3 in OGD/R-induced SH-SY5Y cells.

### IGFBP3 as a functional regulator involved in silenced miR-19a-3p exerted protective roles against OGD/R-induced injury

**Fig. 5:**
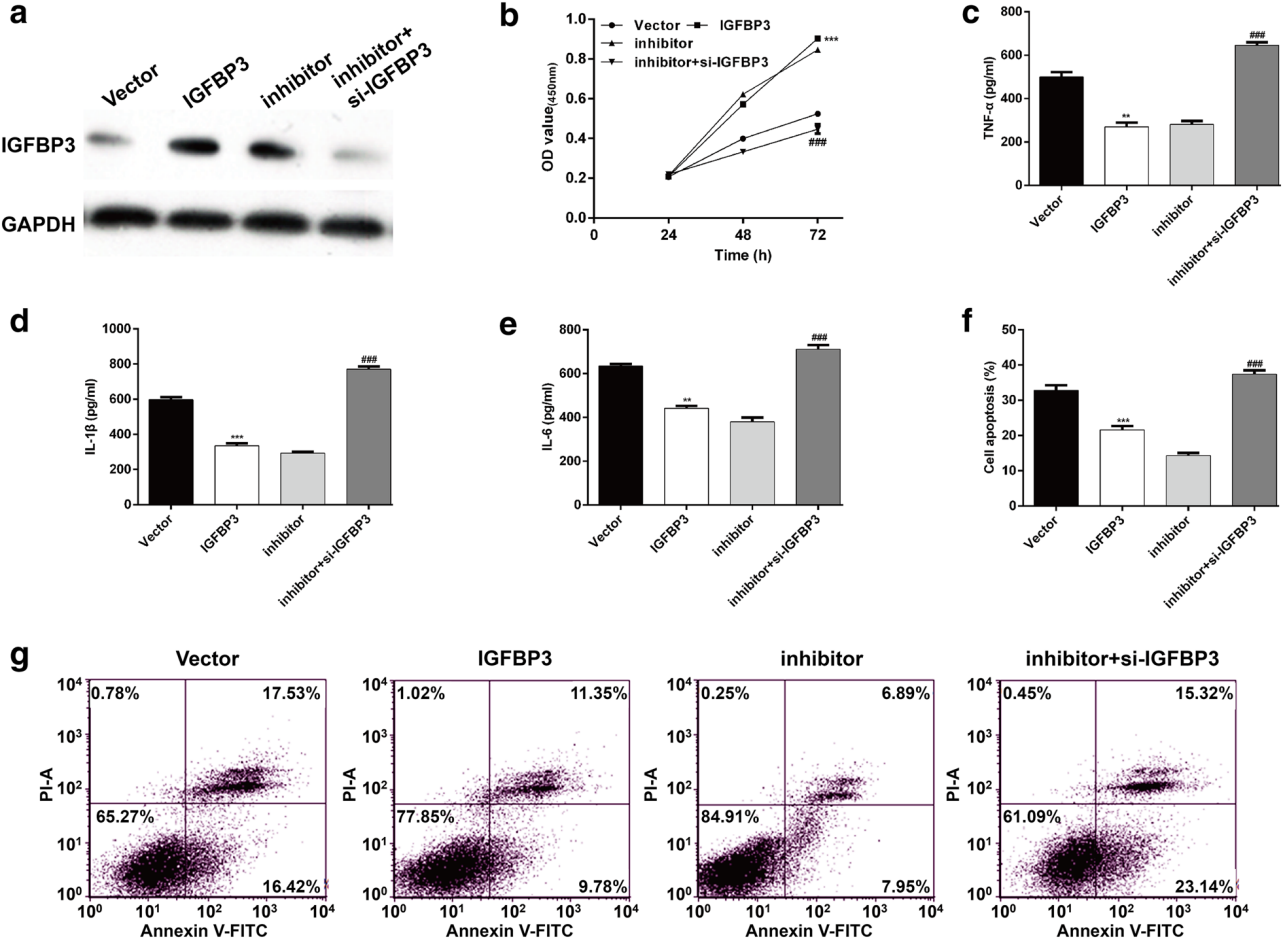
Silenced miR-19a-3p exerted protective roles against OGD/R-induced injury by up-regulating IGFBP3. SH-SY5Y cells were transfected with empty vector, IGFBP3, inhibitor or inhibitor plus si-IGFBP3, respectively, followed by OGD/R exposure. **a** The protein level of IGFBP3 expression was detected by western blot analysis. **b** Cell viability, **c**–**e** pro-inflammatory cytokines and **f**–**g** apoptosis were evaluated in SH-SY5Y cells after the above treatments using CCK-8, ELISA and flow cytometry assays, respectively. The experiments were performed in triplicate and each value represented mean ± SD. **p < 0.01, ***p < 0.001, compared with Vector; ###*p* < 0.01, compared with inhibitor

To further investigate whether IGFBP3 was involved in miR-19a-3p regulating OGD/R-induced injury, SH-SY5Y cells were transfected with empty vector, IGFBP3, inhibitor or inhibitor plus si-IGFBP3, respectively, followed by OGD/R exposure. Western blot analysis confirmed IGFBP3 was up-regulated after sole IGFBP3 or inhibitor transfection, but elevated IGFBP3 expression by inhibitor was abrogated by si-IGFBP3 transfection (Fig. 5a). Results from CCK-8 assay showed that IGFBP3 overexpression significantly improved cell viability in OGD/R induced cells. However, IGFBP3 knockdown obviously reversed the effects of miR-19a-3p inhibitor on cell viability (Fig. 5b). On the contrary, ELISA assay (Fig. 5c–e) and flow cytometry assay (Fig. 5f–g) further demonstrated that the levels of pro-inflammatory cytokines (TNF-α, IL-1β, and IL-6) and cell apoptotic rate were significantly decreased by IGFBP3 overexpression and reduced levels of these pro-inflammatory cytokines and apoptosis by inhibition of miR-19a-3p was remarkably reversed by IGFBP3 knockdown in SH-SY5Y cells after OGD/R exposure. These findings suggest that inhibition of miR-19a-3p exerted protective roles against OGD/R-induced injury might through up-regulating IGFBP3.

## Discussion

Neuroinflammation and apoptosis occupy a crucial role in the complicated pathologies that lead to ischemic brain injury and the subsequent reperfusion damage [3–6, 28]. Previous studies have demonstrated that specific mRNA is considered as potential target against I/R injury. In the present study, RT-qPCR showed the expression of miR-19a-3p was rapidly increased in the brain tissue after I/R. Subsequently, rats were given intracerebroventricular injection of miR-19a-3p inhibitor, followed by MCAO treatment. According to the results, inhibition of miR-19a-3p effectively reduced brain infarct size and ameliorated neurological deficits. We also found inhibition of miR-19a-3p significantly decreased the levels of pro-inflammatory cytokines (TNF-α, IL-1β, and IL-6) and apoptosis after I/R injury in vivo. Similarly, miR-19a-3p was identified as key regulator altered in multiple systems atrophy, as a rare neurodegenerative disorder [29]. Through bioinformatics analysis, Eyileten et al. [16] consistently revealed miR-19a-3p might be proposed as a diagnostic and prognostic biomarker in ischemic stroke.

To further confirm the protective role of miR-19a-3p down-regulation against cerebral I/R injury, we constructed the in vitro OGD/R SH-SY5Y model to analyze the effects of miR-19a-3p on cell inflammation and apoptosis. Consistent with most of studies on ischemic stroke, SH-SY5Y has been frequently chosen as the most commonly used tool in five models of ischemia-related injury, including oxygen and glucose deprivation, H_2_O_2_-induced oxidative stress, oxygen deprivation, glucose deprivation and glutamate excitotoxicity because of its human origin, catecholaminergic neuronal properties, and ease of maintenance [30]. Here, we observed miR-19a-3p inhibitor could promote cell proliferation, and suppress the production of pro-inflammatory cytokines (TNF-α, IL-1β and IL-6) and cell apoptosis in OGD/R-induced SH-SY5Y cells. Our results complement nicely with a previous report showing miR-19a-3p inhibited cell proliferation and promoted cell apoptosis in rheumatoid arthritis fibroblast-like synoviocytes [31]. Notably, a recent study by Ge et al. [32] reported that elevated miR-19a-3p promoted cerebral ischemic injury by modulating glucose metabolism and neuronal apoptosis. Different from this, our study focused on the effect of miR-19a-3p on neuroinflammation and apoptosis in OGD/R-induced SH-SY5Y cells. Moreover, we found different cell types may play different roles in brain injury induced by I/R. As demonstrated by Ge et al. [32], the expression level of miR-19a-3p in rat neurons was significant lower than astrocytes, and induction of I/R in vivo in astrocytes or OGD in vitro in neuronal cells significantly induced miR-19a-3p expression. Here, we used SH-SY5Y cells as the most commonly used tool in five models of ischaemia-related injury. In addition, another study showed miR-19a-3p acts as an oncogene in myeloma by promoting cell proliferation/invasion and inhibiting apoptosis [33]. MiR‑19a‑3p plays an important role in pancreatic β cell function by enhancing cell proliferation and inhibiting cell apoptosis [34]. These differences of miR‑19a‑3p exerting regulatory functions on cell apoptosis might be ascribed to different disease background.

Insulin-like growth factor binding proteins (IGFBPs) are a family of proteins binding to insulin-like growth factors, which have been identified as useful prognostic biomarkers in various malignancies [35]. Recently, IGFBP3 has been reported to be associated with ischemic stroke and significantly decreased in studies from Schwab et al. [22], Denti et al. [23] and Johnsen et al. [24]. Our data demonstrated that IGFBP3 is regulated by miR‑19a‑3p at post translational level and is a direct target of miR‑19a‑3p by luciferase reporter assay. The in vitro OGD/R SH-SY5Y model showed IGFBP3 was significantly decreased, which was opposite with miR‑19a‑3p expression. In agreement with our findings, decreased IGFBP3 expression was negatively correlated with miR‑27‑3p level in blood samples drawn from ischemic stroke patients [36]. Moreover, lower plasma concentration of IGF-1/IGFBP3 increased the risk of prevalent and incident dementia [37]. Furthermore, we further found that IGFBP3 overexpression imitated, while knockdown reversed the protective effects of miR-19a-3p down-regulation against OGD/R-induced injury, which further indicates that IGFBP3 acts as a downstream effector in the miR-19a-3p-mediated function of OGD/R SH-SY5Y model. Of course, many other target genes of miR-19a-3p have also been reported, including PITX1 in gastric cancer [38], SOCS3 in pancreatic β cell function [34], and adiponectin receptor2 (ADIPOR2) in cerebral I/R injury [32]. We believed that there are more target genes of miR-19a-3p will be explored and confirmed in cerebral I/R injury based on different molecular mechanisms.

## Conclusions

In conclusion, we reported for the first time that a mild activation of IGFBP3 by inhibition of miR-19a-3p induced neuroprotective role in cerebral I/R injury by suppressing inflammation and apoptosis. This study will further enhance our understanding of the inflammatory and apoptosis mechanism after cerebral I/R injury and also provide a strong experimental basis for targeting IGFBP3 by miR-19a-3p as a potential therapeutic option.

## Data Availability

The data and materials are available under the permission of author.

## Abbreviation

I/R: Cerebral ischemia/reperfusion
MCAO/R: Middle cerebral artery occlusion/reperfusion
OGD/R: Oxygen-glucose deprivation and reperfusion
IGFBP3: Insulin-like growth factor binding protein-3
TTC: 2, 3, 5-Triphenyltetrazolium chloride
TUNEL: Terminal deoxynucleotidyl-transferase-mediated 2′-deoxyuridine 5′-triphosphate nick-end labeling
SDS-PAGE: Sodium dodecyl sulfate–polyacrylamide gel electrophoresis
